# Microplastic contamination in remote mountain lakes of the americas: a baseline assessment from Patagonia and Northern California

**DOI:** 10.3389/ftox.2026.1851245

**Published:** 2026-05-14

**Authors:** María B. Alfonso, Facundo Scordo, Carina Seitz, Andrés H. Arias, Ana C. Ronda, Gian M. Mavo Manstretta, Sudeep Chandra, Gerardo M. E. Perillo, María C. Piccolo

**Affiliations:** 1 Center for Ocean Plastic Studies, Research Institute for Applied Mechanics, Kyushu University, Kasuga, Japan; 2 Reno’s Biology Department, Wildfire Technology Laboratory, and Global Water Center, University of Nevada, Reno, NV, United States; 3 Instituto de Investigaciones en Biodiversidad y Medioambiente (CONICET - UNCOMA), San Carlos de Bariloche, Argentina; 4 Reno’s Biology Department, and Global Water Center, University of Nevada, Reno, NV, United States; 5 Departamento de Geología y Petróleo, Centro Regional Universitario Bariloche (CONICET-UNCo), San Carlos de Bariloche, Argentina; 6 Instituto Argentino de Oceanografía (IADO), Universidad Nacional del Sur (UNS)-CONICET, Bahía Blanca, Argentina; 7 Departamento de Química, Área III Química Analítica, Universidad Nacional del Sur, Bahía Blanca, Argentina; 8 Departamento de Geografía y Turismo, Universidad Nacional del Sur, Bahía Blanca, Argentina; 9 Departamento de Geología, Universidad Nacional del Sur (UNS), Bahía Blanca, Argentina

**Keywords:** atmospheric deposition, fibers, freshwater, mountain lakes, patagonia, remote lakes

## Abstract

Plastic pollution and its effects on freshwater ecosystem function are increasingly recognized, with recent research demonstrating that even remote environments are impacted. Despite growing evidence, the relative importance of local versus large-scale transport processes in delivering microplastics (MPs) to remote mountain lakes remains poorly understood. Surface water samples were collected by net trawling in 15 remote mountain lakes, eleven in the Patagonia region of Argentina and four in Northern California, United States, and analyzed by stereomicroscopy and Raman spectroscopy for MP concentration, shape, size, color, and polymer composition. MPs (≥ 80 µm) were detected at all sites, with concentrations ranging from 0.23 to 2.79 particles m^−3^ (mean: 0.75 ± 0.62 particles m^−3^). No significant difference in MP concentration was observed between regions (U = 12; p = 0.215), despite contrasting socioeconomic contexts. Fibers were predominant (70%), followed by fragments (24%) and films (6%), with most particles measuring less than 1 mm and over half under 0.5 mm. Ten polymer types were identified, with polyester (PES) and polyethylene terephthalate (PET) most common, primarily as fibers. No significant relationships were found between MP concentration and watershed characteristics, proximity to urban areas, public access, or altitude. The consistent MP signature across these remote lakes, regardless of morphology, accessibility, or geographic region, suggests that diffuse, large-scale transport mechanisms such as atmospheric deposition and watershed connectivity are more influential than localized point sources in remote freshwater systems. However, certain lakes near urban infrastructure or camping areas exhibited higher MP concentrations, indicating that localized anthropogenic influences may still contribute in specific cases. These findings underscore the extensive impact of MP pollution and highlight the need for coordinated mitigation strategies at local, regional, and global scales.

## 1 Introduction

The continuous increase in plastic production, use, and mismanagement has made microplastic (MP, particles <5 mm) pollution ubiquitous in the environment (Bergmann et al., 2019), from remote, pristine areas to the human body (Thompson et al., 2024). To date, many studies on aquatic systems have documented their presence, accumulation (Dusaucy et al., 2021; Chen et al., 2024), and impact on organisms (Huang et al., 2021). Additionally, MPs can act as carriers of toxic chemicals, causing risks through water contamination and bioaccumulation in the food chain (Wiesinger et al., 2021; Beiras et al., 2021; Yu et al., 2024).

Lakes face escalating environmental pressures from human activities and tourism, which accelerate biodiversity loss and degrade water quality, including through MP contamination (Weyhenmeyer et al., 2024). This vulnerability is pronounced because lakes, while containing only 0.8% of the world’s non-frozen freshwater, are disproportionately affected by anthropogenic impacts. Their extensive and complex shorelines, approximately four times longer than the global ocean coastline (Messager et al., 2016), create a critical land–water interface that intensifies exposure to development, runoff pollution, habitat alteration, and recreational use. These factors collectively threaten the environmental health of freshwater ecosystems. Previous studies indicate that lakes with low depth-to-area ratios or high watershed-to-lake area ratios often exhibit higher MP concentrations, likely due to increased particle accumulation from watershed runoff, adjacent human activity, or fluvial inputs (Grbic et al., 2020; Wang et al., 2023; McIlwraith et al., 2024).

Remote lakes are often regarded as sentinels of climate change because their hydrology, mixing regimes, and temperature are highly sensitive to temperature fluctuations, glacial retreats, and ecological changes within their basins (Adrian et al., 2009; Williamson et al., 2009). Lakes in arid and semiarid regions are typically isolated and particularly vulnerable to climate change, often possessing small surface areas that may shrink further under extreme climatic conditions (Scordo et al., 2025). These hydrological constraints can enhance the retention and concentration of MPs compared to larger or more hydrologically dynamic lakes (Hueso-Kortekaas et al., 2025). Shallow lakes with limited water volume may function as efficient MP sinks due to reduced dilution capacity and restricted hydrological dispersion, resulting in higher surface concentrations than deeper systems (Alfonso et al., 2020b; Nava et al., 2023). Despite their ecological significance, water sources in remote and protected areas remain understudied due to logistical challenges and high sampling costs (Smits et al., 2025). Therefore, investigating MP pollution in remote lakes is essential for understanding its broad impacts on biodiversity and water quality.

Plastic composition in lakes and particularly in remote or pristine freshwater environments tends to be dominated by synthetic fibers rather than fragments or films (e.g., Negrete Velasco et al., 2020; Dusaucy et al., 2021; McIlwraith et al., 2024). This pattern has been attributed to the widespread use of synthetic textiles, the ability of fibers to be easily transported through wind and water, and their persistence in the environment. Fibers are also more likely to enter remote systems *via* atmospheric deposition (Brahney et al., 2020) or diffuse sources such as runoff (McIlwraith et al., 2024) rather than through direct point discharges from sewerage systems and wastewater treatment plants, which are generally observed in lakes closer to urban centers (Xia et al., 2022) even if they are alpine (Todeschini et al., 2025). As a result, fiber-dominated MP profiles may reflect low-intensity but widespread human activity rather than point-source pollution in remote lakes. Understanding the prevalence of particular particle shapes and polymer types is key to identifying pollution sources and transport mechanisms in remote regions.

Despite increasing evidence of MP contamination in remote freshwater systems, the relevance of morphological and hydrological predictors identified in urbanized lake systems, such as watershed-to-lake area ratio, lake depth, and proximity to human activity, remains uncertain in truly remote settings characterized by low and diffuse anthropogenic pressure. Additionally, most studies on remote lakes are geographically limited to single regions, leaving unresolved whether MP contamination patterns are consistent across contrasting hemispheres and socioeconomic contexts or are primarily influenced by local environmental conditions.

The present study examines MP concentrations and characteristics in 15 remote mountain lakes, eleven in the Andean Range (Argentine Patagonia, southern South America) and four in the Klamath Mountains (northern California, United States), to assess the influence of watershed features and varying levels of anthropogenic activity on MP contamination in these ecologically significant yet understudied freshwater systems. Although both regions are mountainous, they differ substantially in economic development, population density, and land use (see Section 2.1). The research specifically addresses: (1) whether MP concentrations are elevated in lakes with greater human influence, such as proximity to cities or recreational facilities; (2) whether the watershed-to-lake area ratio predicts MP contamination in remote settings; (3) whether lake depth affects MP accumulation profiles; and (4) whether altitude correlates with lower MP concentrations. By integrating morphological, environmental, and polymer data from two contrasting regions, this study provides baseline information on MP pollution in remote mountain lakes of the Americas and evaluates the relative importance of local *versus* large-scale processes in determining MP contamination in remote freshwater systems.

## 2 Materials and methods

### 2.1 Study sites and sample collection

**FIGURE 1:**
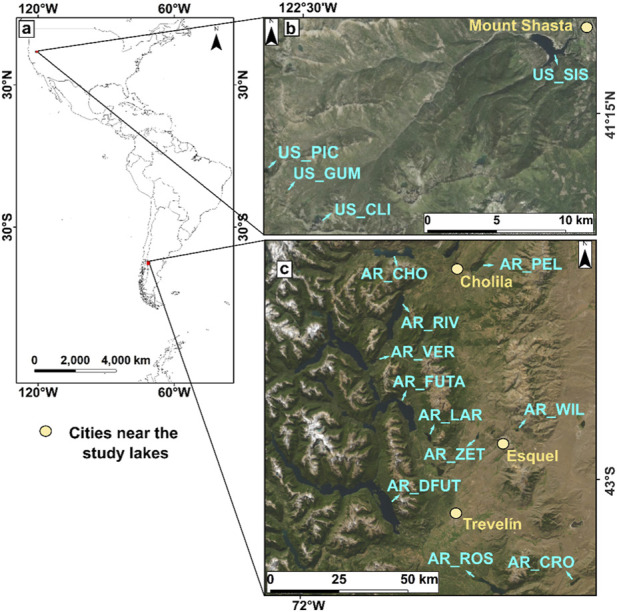
Geographical location of the **(a)** remote mountain study areas from America, including the location of the lakes and reservoirs studied within **(b)** the United States of America in northwestern California and **(c)** the center and west of Argentine Patagonia. Arrows indicate lakes and reservoir locations. Acronyms: Lake Rosario (AR_ROS), Futaleufú Reservoir (AR_DFUT), Lake Verde (AR_VER), Lake Futalaufquen (AR_FUTA), Lake Cronómetro (AR_CRO), Lake Zeta (AR_ZET), Lake Willimanco (AR_WIL), Lake Larga (AR_LAR), Lake Rivadavia (AR_RIV), Lake Pellegrini (AR_PEL), Lake Cholila (AR_CHO), Lake Siskiyou (US_SIS), Lake Cliff (US_CLI), Lake Picayune (US_PIC), and Lake Gumboot (US_GUM).

To compare the presence and characteristics of MPs in remote mountain lakes and reservoirs of the Americas, two survey campaigns were developed during the summer season in eleven lakes from Argentina (AR; December 2019) and four lakes from the United States of America (US; August 2020) (Figure 1; Supplementary Tables S1, S2). The Argentinian sites are in the Futaleufú River Basin in the Patagonia Andean Range region and include Lake Rosario (AR_ROS), Futaleufú Reservoir (AR_DFUT), Lake Verde (AR_VER), Lake Futalaufquen (AR_FUTA), Lake Cronómetro (AR_CRO), Lake Zeta (AR_ZET), Lake Willimanco (AR_WIL), Lake Larga (AR_LAR), Lake Rivadavia (AR_RIV), Lake Pellegrini (AR_PEL) and Lake Cholila (AR_CHO). The US sites are situated in the Klamath Mountains of Northern California and include Lake Siskiyou (US_SIS), Lake Cliff (US_CLI), Lake Picayune (US_PIC), and Lake Gumboot (US_GUM). The waterbodies presented a wide range of morphometric and geographic features, with surface area values ranging from 0.03 to 92 km^2^, maximum water depth between 4.6 and 168 m, and altitudes between 480 and 1860 m. a.s.l. (Supplementary Tables S2).

The lakes in both regions exhibit distinct hydrological features (see Supplementary Section S1), forming part of a complex mountain hydrological system, some of which are located within natural protected areas, each with varying degrees of use and management. All the lakes are in low-populated areas (2,000 to 37,000 residents); however, tourism (e.g., boating, camping, fishing, skiing, and hiking activities) is important in both regions. Furthermore, US_SIS and AR_DFUT are reservoirs. Mount Shasta, with around 3,200 residents (U.S. Census Bureau, 2021), is the closest city to the United States lakes. US_SIS is the nearest (4 km) waterbody to Mount Shasta and receives the most tourism, while the rest of the lakes (located more than 25 km away from Mount Shasta) are part of the Pacific Crest Trail (the most visited hiking path along the crest of the Pacific Mountain ranges). The closest cities to the Argentinian lakes are Cholila (2,831 residents), Esquel (37,261 residents), and Trevelin (10,812 residents) (INDEC, 2022). AR_ZET and AR_PEL are less than 6 km from Esquel and Cholila, respectively, while the other lakes are located over 10 km away from the nearest cities.

Surface water samples were obtained by trawling a plankton net with mesh sizes of 38 µm in Argentina and 80 µm in the United States, towed by boat at approximately two knots (trawling method; UNEP, 2020). The difference in mesh sizes was based on equipment availability during each field campaign. Therefore, only particles ≥80 µm were included in the analysis to ensure comparability across sites. Whenever feasible, sampling locations were primarily near the lake outlet, with trawling distances ranging from 0.4 to 1 km, adjusted based on water conditions to prevent net clogging. Navigation parameters, including speed and distance, were monitored using a Garmin echo sounder with GPS (model GPSMAP® 721). Water volume was estimated by multiplying the trawling distance by the submerged area of the net opening (Supplementary Tables S1).

### 2.2 Morphometric characterization of lakes and their watershed

The watershed area of the lakes was determined using Global Mapper V-15 (http://GlobalMapper.com), which utilized the NASA SRTM (Shuttle Radar Topography Mission) Digital Elevation Model (DEM) to assess the sub-catchment areas for each lake. The DEM has a pixel spatial resolution of 90 × 90 m and a 1 m vertical resolution. To assess the shoreline length and lake area, Landsat 8 Operational Land Imager (OLI; bands 5, 6, and 4) data downloaded from Google Earth Engine was used (https://developers.google.com/earth-engine/datasets/catalog/landsat). Landsat Collection 2 - Level two product that provides surface reflectance data with corresponding atmospheric, radiometric, and geographic corrections was used. RGB (near-infrared; far-infrared; red; Landsat 8 OLI: bands 5-6-4) band stack was used to distinguish water bodies from other land types. This band composition effectively differentiates water from various land covers (Horning, 2004) and has been successfully implemented in other lakes in Patagonia (Scordo et al., 2025). The lake area was obtained by implementing a supervised classification method (maximum likelihood method) based on a defined region of interest (ROI) on the RGB combinations of each image, followed by the vectorization of the defined layers. Finally, the raster calculation tool from ArcGIS 10.0 was used to calculate the lake surface area. The distance from each lake to the closest urban center was measured linearly using the basemap in the ArcGIS software, the shapes of the lakes, and the closest cities. Information regarding access to the lakes was determined during fieldwork. The altitude was measured using a GPS Garmin eTrex Vista (datum WGS84). The lake’s maximum depth was obtained from published literature (Supplementary Tables S2; Quirós and Drago, 1985; U.S. Forest Service, 2014).

Lakes were categorized based on physical and environmental factors to examine if these variables influence the MP concentrations (particles m^-3^). Specifically, lakes were grouped by “lake area and depth” (Larea/Depth) into three categories: Small/Shallow (<1 km^2^ area and <20 m depth), Medium/Intermediate (1–20 km^2^ area and 20–100 m depth), and Large/Deep (>20 km^2^ area and >100 m depth). To evaluate human influence, “distance from urban centers” and “public access” categories were created, classifying lakes as Close (<10 km), Moderate (10–30 km), or Far (>30 km) from the nearest town, and indicating whether access was Public (access) or Restricted (No access). Additionally, an “altitude” category (Altitude) was used to capture environmental variability, grouping lakes as Low (<1,500 m. a.s.l.) or High (>1,500 m. a.s.l.) altitude (Supplementary Tables S2).

### 2.3 Sample processing and analysis

To determine the abundance and characteristics of MPs, each sample was transferred to a clean glass beaker for organic matter digestion with 30% H_2_O_2_ on a temperature-controlled heating plate at 60 °C for up to 10 h to prevent damage to plastic particles (Michida et al., 2019; Alfonso et al., 2020a). After digestion, the samples were filtered using 1.2 µm pore size Whatman fiberglass filters and a vacuum pump. The filters were then stored in clean Petri dishes for subsequent particle counting and characterization. The retained particles were analyzed under a Nikon SMZ1500 stereomicroscope equipped with an As One digital camera PCS500 and its measuring software. Each potential MP was photographed and classified by color, shape (bead, fiber, line, film, fragment), and size using Feret’s maximum diameter (µm) (Michida et al., 2019).

Identifying MPs through microscopy may produce false positives, with natural materials being incorrectly classified as plastics (Hidalgo-Ruz et al., 2012). To verify polymer composition, a random subsample of particles from each site was analyzed to confirm whether they were plastic polymers. Suspected plastic particles were mounted onto glass slides with double-sided adhesive tape using tweezers for further analysis. A total of 100 particles, representing 18% of the overall particle count, were analyzed, which is sufficient according to current guidelines (De Frond et al., 2023; Hermsen et al., 2018).

Each particle was analyzed using Raman microscopy (LabRAM HR Evolution, Horiba, France). Spectra were measured using a 50× long working distance (LWD) objective at a wavelength of 785 nm, with a 25% laser filter focused on the sample surface. The confocal pinhole was set to 100 μm, and a 300 groove/mm grating was used, covering the Raman range of 150–3,500 cm^-1^. The Raman spectrum was measured with a 10-s exposure time and ten accumulating scans at two points on each sample.

For polymer identification, the obtained spectra (as CSV files) were compared to the polymer database of the open-source software Open Specy (Cowger et al., 2021). Particles of synthetic or semi-synthetic origin or processed natural materials resulting from human activity that were not confirmed as plastic polymers through spectroscopic analysis were classified as anthropogenic particles. This category includes particles with artificial colors, uniform morphology, or textures indicative of industrial processing (e.g., dyed cellulose fibers, rayon, viscose, polyester-cotton blends). Since the indigo blue pigment spectrum overlaps with the signal of potential plastic polymers (González-Pleiter et al., 2020), particles showing this spectrum were classified and recorded as indigo blue. The remaining particles were classified as not plastic. Final MP concentrations were adjusted per country according to the percentage of positive plastic polymer and anthropogenic particle identifications.

### 2.4 Quality assurance and quality control

Negative control samples (N = 3) were prepared with filtered distilled water and analyzed simultaneously with environmental samples to detect potential contamination during sample processing. A total of three indigo-blue fibers were found across all blank samples (median value = 1). The number of particles for each polymer type identified in the negative controls (indigo blue = 1) was subtracted from the environmental sample counts to account for background contamination, following common practice in microplastic research (Rotchell et al., 2023). All working surfaces were cleaned with 70% ethanol, and lab materials were washed and rinsed with distilled water before use. Cotton laboratory coats and nitrile gloves were worn during all procedures. Laboratory personnel circulation was minimized during sample processing to reduce contamination exposure. Glass and metal equipment were used throughout, and all reagents were vacuum filtered (GF/C, 1.2 µm) before use. All samples were covered with aluminum foil and processed under a fume hood to minimize contamination during processing.

### 2.5 Data analysis

A lower size limit of ≥80 µm was consistently applied for all lakes to ensure data comparability across sites. Lakes were classified into concentration categories using quantile thresholds derived from the distribution of MP concentrations (particles m^-3^), resulting in three groups: low (<Q1), medium (≥Q1 and ≤ Q3), and high (>Q3) concentration. Normality was first assessed using the Shapiro-Wilk test. As data deviated from a normal distribution, non-parametric statistical methods were applied for all subsequent analyses. The Mann-Whitney U test was used to compare MP concentrations between countries and between categories of altitude and public access. The effect of L_area_/Depth and distance to urban centers on MP concentration was assessed using the Kruskal–Wallis test. A summary of the statistical results is presented in Supplementary Tables S3. Spearman correlation analysis was performed to examine potential relationships between MP concentrations and watershed or lake morphological variables, including lake area, maximum depth, watershed-to-lake area ratio, altitude, and distance to the nearest urban center (Supplementary Tables S4). All analyses were conducted with a statistical threshold of p < 0.05 using R (version 4.5.1, R Core Team, 2025).

## 3 Results

### 3.1 Microplastics concentrations

**FIGURE 2:**
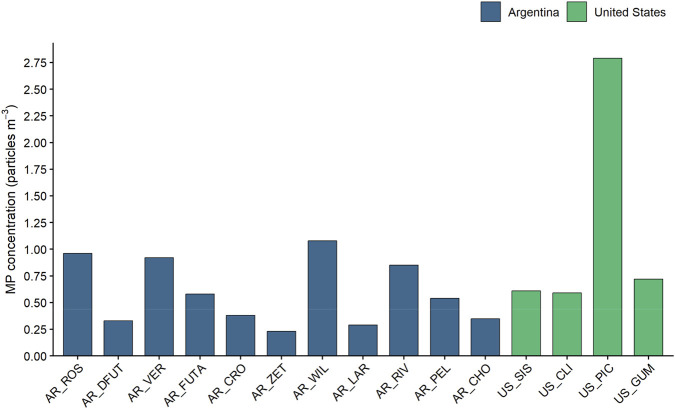
Bar graphs showing microplastic concentration values (particles m^-3^) for all the study sites in the United States and Argentina (only particles ≥80 μm).

To characterize MP contamination across the 15 remote mountain lakes, surface water samples were analyzed and MP concentrations compared between regions and concentration categories. MPs were present in all 15 remote lakes sampled across both regions. Abundance values ranged between 0.23 particles m^-3^ (AR_ZET) and 2.79 particles m^-3^ (US_PIC) (Figure 2; Supplementary Tables S1), with a mean concentration of 0.75 ± 0.62 particles m^-3^ across all sites. Based on the quantile classification method, lakes with low concentrations (<0.37 particles m^-3^) included AR_ZET, AR_LAR, AR_DFUT, and AR_CHO, all located in Argentina. The medium concentration group (≥0.37 and ≤0.89 particles m^-3^) comprised lakes from both regions, including AR_CRO, AR_PEL, AR_FUTA, AR_RIV, US_CLI, US_SIS, and US_GUM. The high concentration group (>0.89 particles m-3) consisted of AR_VER, AR_ROS, AR_WIL, and US_PIC. When analyzed by country, mean concentrations were 1.18 ± 1.08 particles m^-3^ in the US and 0.59 ± 0.31 particles m^-3^ in AR, with no statistically significant difference between regions (U = 12; p = 0.215).

### 3.2 Shape, size, color and polymer characterization of MPs

**FIGURE 3:**
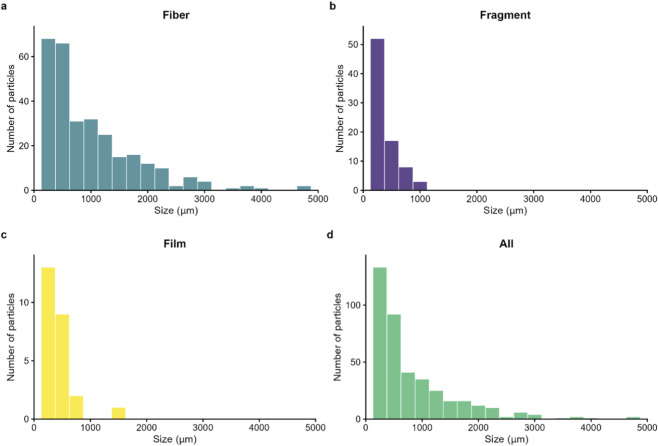
Size frequency distribution (μm) for **(a)** Fiber, **(b)** Fragment, **(c)** Film and, **(d)** all the particles included in this study.

MP particles were characterized by shape, size, color, and polymer type to identify potential sources and transport mechanisms. Fibers were the predominant MP shape across all lakes, representing 70% of occurrences, followed by fragments (24%) and films (6%). Shape composition differed slightly between regions: AR showed 75% fibers, 23% fragments, and 2% films, while US lakes had 63% fibers, 25% fragments, and 12% films. No primary MPs such as pellets or microbeads were recorded in any lake. Size distributions revealed that 73% of particles across all morphologies measured under 1 mm, with 53% under 0.5 mm and 29% under 0.3 mm (Figure 3). Overall dimensions ranged from 80 to 4,802 µm. When broken out by shape, 87% of fragments and 72% of films were under 0.5 mm, whereas fibers exhibited a broader size span: only 40% fell below 0.5 mm, but 63% were under 1 mm and 79% under 1.5 mm. In terms of color, black and blue particles were predominant across all lakes, mainly within the fiber category, followed by red, orange, and yellow particles (Figure 5).

**FIGURE 4:**
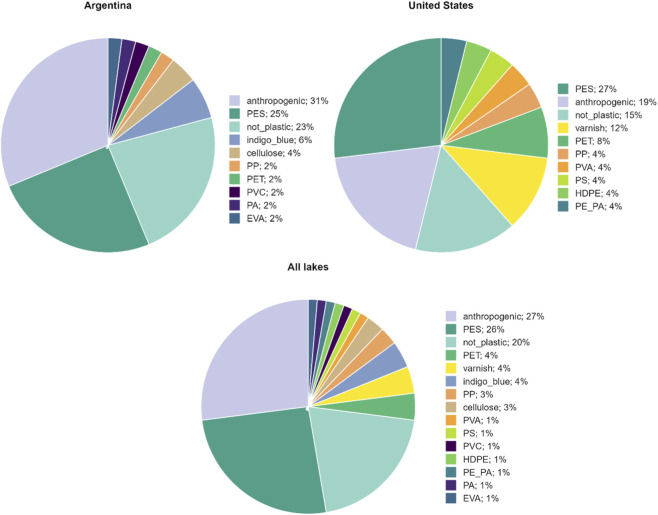
Comparative pie charts illustrating the relative abundance (%) of different plastic polymer types identified in each country and for all the lakes.

A total of 10 polymer types were identified across all lakes, including polypropylene (PP), polyester (PES), polyethylene terephthalate (PET), polyvinyl alcohol (PVA), polystyrene (PS), polyvinyl chloride (PVC), high-density polyethylene (HDPE), polyethylene-polyamide (PE-PA), polyamide (PA), and ethylene-vinyl acetate (EVA), along with varnish and the textile dye indigo blue. In AR lakes, anthropogenic particles predominated (31%), followed by PES (25%), indigo blue (6%), and others including PVC, PP, PET, PA, and EVA (Figure 4). In US lakes, PES (27%) and PET (8%) were dominant, followed by anthropogenic particles (19%) and varnish (12%) among others (Figure 4).

**FIGURE 5:**
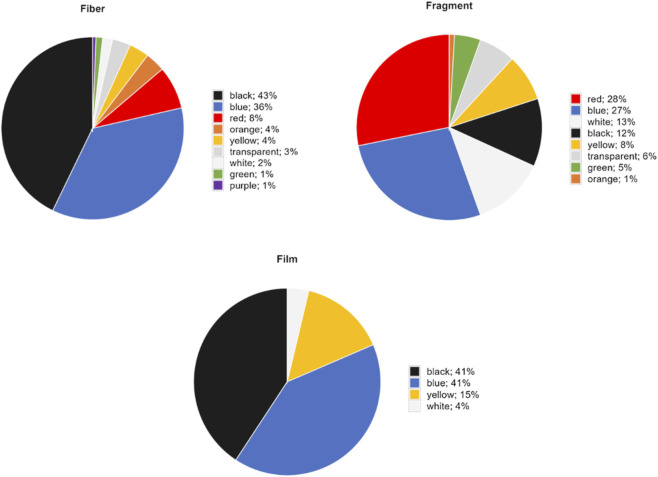
Proportional distribution of MP colors by particle shape across the entire study.

### 3.3 Influence of morphological and anthropogenic variables on MP concentration

**FIGURE 6:**
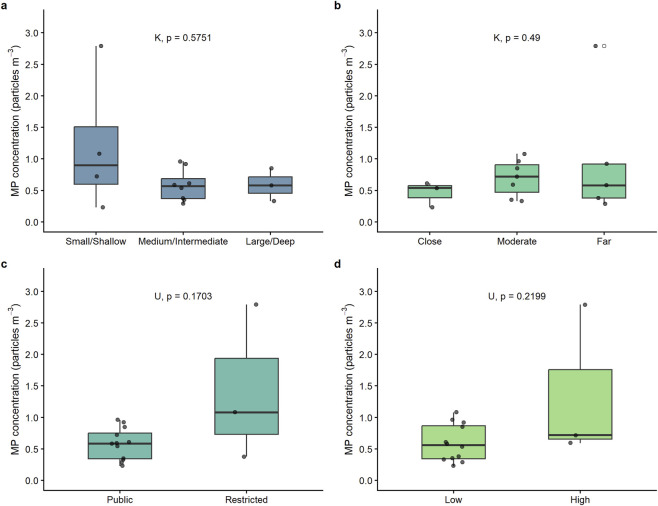
Box plots showing MP concentrations (particles m^-3^) across all studied lakes, categorized by: **(a)** lake area-to-maximum depth ratio (L_area_/Depth), **(b)** distance to urban centers, **(c)** public access, and **(d)** altitude. Corresponding p-values are shown for each comparison based on Kruskal–Wallis (K) or Mann-Whitney U (U) tests, as appropriate.

To evaluate whether local watershed features and anthropogenic pressure explain MP variability across lakes, a series of non-parametric statistical tests and Spearman correlations were performed. All p-values exceeded the 0.05 significance threshold, indicating no statistically significant differences in MP concentrations for any of the variables analyzed (Figure 6; Supplementary Tables S3). Spearman correlation analysis found no significant relationships between MP concentration and lake area, maximum depth, watershed-to-lake area ratio, altitude, or distance to the nearest urban center across the full dataset (Supplementary Tables S4). Although statistical significance was not reached, lakes classified as Small/Shallow exhibited higher mean MP concentrations than Medium/Intermediate and Large/Deep lakes (Figure 6a). Lakes farther from urban centers did not show significantly lower MP concentrations compared to those closer to urban areas, nor did lakes with restricted public access show lower concentrations than those with public access (Figures 6b,c). Low-altitude lakes had MP concentrations similar to those at high altitude (Figure 6d).

## 4 Discussion

### 4.1 Microplastic concentrations in context

MPs were detected in all 15 remote lakes sampled, with a mean concentration of 0.75 ± 0.62 particles m^-3^ and no significant difference between regions (U = 12; p = 0.215), despite their contrasting socioeconomic contexts. These results are consistent with the expectation that remote, low-populated lake systems generally exhibit low MP contamination levels. MP pollution in lakes worldwide is considered relatively low when concentrations are below one particle m^-3^ (Nava et al., 2023), and most lakes in both regions fell within or near this threshold.

Comparisons with other studies remain limited due to difficult survey access to remote areas and methodological differences, particularly in mesh sizes used for particle collection (typically around 300 µm) or units used to express results (e.g., particles km^-2^). Compared to previous studies using similar methodologies and particle size ranges in Patagonian lakes, the concentrations observed here were within a comparable range. Reported values from the AR region include 0.29 to 1.72 particles m^-3^ for particles >80 µm in nine Patagonian lakes (Alfonso et al., 2020a) and 0.89 to 3.3 particles m^-3^ for particles >100 µm in six Patagonian lakes (Buteler et al., 2023). Studies in sparsely populated or mountain lakes of the United States have reported similar concentrations despite using larger mesh sizes, which likely leads to underestimation of particle abundance. For instance, Xiong et al. (2022) found 1.69 particles m^-3^ in Lake Flathead using a 330 μm mesh, and Baldwin et al. (2020) reported concentrations ranging from 0.44 to 1.99 particles m^-3^ in Lakes Mead and Mohave using a 100 µm mesh. Studies using trawling nets (∼300 µm) in semi-remote Canadian lakes reported values between 0.1 and 0.6 particles m^-3^, while water pump sampling yielded 0.03 to 1.3 particles m^-3^ (McIlwraith et al., 2024), consistent with the present study findings.

In contrast, studies worldwide using similarly fine mesh sizes have reported substantially higher MP concentrations: 40.6 particles m^-3^ in Lake Guaíba, Brazil (Bertoldi et al., 2021); 240 to 1800 particles m^-3^ in Lakes Poyang and Hong, China (Li et al., 2019); and 5,900 particles m^-3^ in Red Hills Lake, India (Gopinath et al., 2020). These lakes are situated in densely populated and urbanized regions receiving significant inputs from urban runoff, domestic wastewater, and industrial discharges. It is worth noting that many freshwater studies use manta or plankton nets with mesh sizes of ∼300 μm, which likely leads to underestimation of particle abundance in remote systems such as those examined here.

Although the MP concentrations recorded in this study are generally low, the potential ecological implications for resident biota should not be overlooked. The small size of most particles (>53% under 0.5 mm) and the dominance of fibers increase their amenability to ingestion by a wide range of freshwater organisms, including filter feeders, zooplankton, and small fish (Huang et al., 2021). Synthetic fibers in particular have been shown to cause physical damage to the digestive tract, reduce food intake, and act as vectors for toxic chemical additives in aquatic invertebrates and vertebrates (Beiras et al., 2021; Yu et al., 2024). The presence of PES and PET particles, which can carry and release plasticizers and other additives, is of additional concern for resident biota in these otherwise pristine systems (Yu et al., 2024). While a formal risk assessment is beyond the scope of this study, the ubiquitous presence of MPs in remote lakes with no significant local sources underscores the need to consider baseline exposure levels even in protected freshwater ecosystems.

### 4.2 Shape, size, color and polymer composition

Previous studies have suggested that remote lakes tend to be dominated by synthetic fibers rather than fragments or films, with fiber dominance attributed to long-range atmospheric transport and diffuse inputs rather than direct point-source discharges (Negrete Velasco et al., 2020; Dusaucy et al., 2021; McIlwraith et al., 2024). The results of this study are consistent with this pattern. Fibers dominated MP composition across all lakes (70%), with similar proportions in both regions, and most particles were smaller than 1 mm. These proportions are consistent with previous global studies (Dusaucy et al., 2021; Chen et al., 2024) and with findings from remote lakes specifically, where fibers are often the dominant shape (Zhang et al., 2024). A recent global study (Nava et al., 2023) reported that MP shape composition can vary with lake morphometry, where fibers tend to dominate in small, shallow lakes, while fragments are more prevalent in larger, deeper systems. The dominance of fibers across all lakes in this study, regardless of depth or surface area, suggests that in remote freshwater systems fiber prevalence may be less related to lake morphometry and more indicative of long-range transport and persistent diffuse inputs.

Fibers in lakes are often associated with urban sources such as wastewater discharge and laundering activities (De Falco et al., 2020). In remote, sparsely populated regions, direct urban inputs are minimal or absent. Instead, synthetic fibers from outdoor clothing and textiles used during recreational and camping activities may contribute to MP fiber pollution. However, attributing fiber presence to a single source oversimplifies the complexity of MP contamination in these environments. Multiple diffuse and interconnected pathways, including atmospheric deposition, long-range transport, runoff from intermittent human activity, and legacy pollution through resuspension of previously deposited fibers, can introduce synthetic fibers into remote lakes, underscoring the necessity for mitigation strategies at local, regional, and global scales (Thompson et al., 2024).

The predominance of small fragments also is consistent with previous studies where dry and wet deposition of MPs prevail in remote high-altitude settings and the photo-oxidative breakdown of particles under intense UV exposure (Klein and Fischer, 2019; Sørensen et al., 2021; Ohno and Iizuka, 2023). At elevation, colder temperatures and significant snowfall increase atmospheric capture of MPs, which become entrained in the snowpack and are later released during melt (Ohno and Iizuka, 2023; Karapetrova et al., 2024). Notably, analyses in high-altitude (>2000 m) snow pits from California have recovered 35–913 particles per liter of meltwater, predominantly fragments under 100 μm, illustrating the importance of atmospheric pathways in mountain MP contamination (Karapetrova et al., 2024).

Regarding polymer composition, PES and PET dominated in both regions and were primarily associated with fiber shape, consistent with previous global studies (Chen et al., 2024). Anthropogenic particles, including dyed cellulose fibers, rayon, and textile blends not confirmed as plastic polymers by spectroscopy, were also abundant, particularly in AR lakes (31%), reflecting the widespread use of semi-synthetic textiles in outdoor settings. The presence of indigo blue, a common textile dye that interferes with polymer identification by Raman spectroscopy, further supports the anthropogenic origin of a significant fraction of the particles identified (Turner et al., 2019). Other polymers detected in lower proportions, including PP, PVC, HDPE, PE-PA, PA, EVA, and varnish, likely reflect diverse incidental inputs from recreational equipment, fishing gear, and infrastructure materials. The prevalence of both dark and bright-colored polyester and PET fibers suggests abrasion of outdoor apparel and technical gear such as jackets, ropes, and backpacks as a key source, as documented in alpine lake studies (Pastorino et al., 2023; Lasota et al., 2023; Kiełtyk et al., 2024). Notably, no primary MPs such as pellets or microbeads were recorded in any lake, further supporting the dominance of diffuse and recreational sources over point-source inputs in these remote systems.

### 4.3 Influence of morphological and anthropogenic variables

One of the central questions of this study was whether local watershed features and anthropogenic pressure predict MP concentrations in remote mountain lakes, as has been suggested for more urbanized systems (Grbic et al., 2020; Wang et al., 2023; McIlwraith et al., 2024). The absence of statistically significant relationships between MP concentration and any of the variables analyzed, including lake area, maximum depth, watershed-to-lake area ratio, altitude, distance to urban centers, and public access, suggests that this is not the case in truly remote settings. Together with the particle size and shape signatures discussed in Section 4.2, these findings point to the possible role of regional to global processes, such as hydrological connectivity or atmospheric deposition, in transporting MPs to pristine environments, consistent with previous studies in remote areas (Jiang et al., 2019; Brahney et al., 2020; Grbic et al., 2020).

Despite the overall lack of significant differences, some patterns within the data offer additional insights. Although overall statistical significance was not reached, Small/Shallow lakes showed higher mean MP concentrations than Medium/Intermediate and Large/Deep lakes, suggesting that reduced water volume may limit dilution capacity and result in localized accumulation of MPs (Alfonso et al., 2020b). These environments may function as sinks for MPs due to limited hydrological dispersion, increasing their vulnerability to contamination. This is particularly relevant for shallow terminal lakes experiencing rapid drying globally (Wang et al., 2018) and in Patagonia specifically (Scordo et al., 2025).

Lakes farther from urban centers did not show significantly lower MP concentrations compared to those closer to urban areas, nor did lakes with restricted public access show lower concentrations than those with public access (Figures 6b,c). This suggests that proximity to urban areas and accessibility do not determine MP levels at the regional scale in these remote lakes. However, notable differences were observed between two small and shallow lakes in Argentina, AR_ZET and AR_WIL, despite their similar morphometry and altitude. AR_WIL recorded the highest MP concentration among the Argentine lakes (1.08 particles m^-3^), which may be partially explained by its location along a direct line from the solid waste management facility serving Esquel and Trevelin, as well as its proximity to a frequently used national highway. In contrast, AR_ZET showed a much lower concentration (0.23 particles m^-3^), possibly influenced by its higher elevation relative to the city (770 m vs. 560 m) and its more protected location surrounded by high terrain. AR_ZET was predominantly composed of fibers (91%), suggesting fewer diverse sources, whereas AR_WIL presented a more mixed composition with 42% fragments and 58% fibers, indicating multiple input pathways. Other Argentine lakes with higher MP abundances included AR_ROS (0.96 particles m^-3^), which has numerous private docks and popular coastal camping areas, and AR_RIV (0.85 particles m^-3^), located in Los Alerces National Park with several camping sites and popular boating areas, whose main tributary crosses a small urban area. The outlet of AR_RIV connects to AR_VER (0.92 particles m^-3^), situated in a highly frequented tourist area with camping, boating, and lodging facilities, and which has the highest watershed-to-lake area ratio among all sites, suggesting a significant influence of runoff and downstream concentration.

Taken together, these lake-level patterns suggest that while large-scale transport mechanisms dominate MP delivery to remote systems, localized anthropogenic influences can modulate concentrations at specific sites. The absence of an altitudinal gradient further reinforces that remoteness alone does not protect against MP contamination. Atmospheric transport mechanisms and local land use practices likely play a more dominant role, consistent with emerging evidence on atmospheric deposition as a global pathway for MPs in remote environments (Allen et al., 2019; Brahney et al., 2020; Parolini et al., 2021). These results emphasize the complexity of MP distribution in remote mountain areas, where multiple factors likely interact to influence concentrations.

### 4.4 Limitations

Several limitations of this study should be acknowledged. Only one surface water sample was collected per lake, which prevents assessment of temporal variability or seasonal dynamics in MP abundance and composition. The absence of concurrent meteorological and atmospheric deposition data limits mechanistic interpretation of MP transport pathways, and the asymmetric sample sizes between regions (n = 11 AR vs. n = 4 US) constrain direct statistical comparisons between countries. Additionally, the use of different mesh sizes between campaigns (38 µm in AR vs. 80 µm in US), while addressed through a consistent analytical lower size limit of ≥80 μm, may have introduced some differences in sampling efficiency not completely captured by size-based harmonization. In particular, the 80 µm lower size limit may have resulted in the underrepresentation of smaller fragments and films, which are more readily captured by finer meshes. Future studies should address these limitations *via* repeated sampling across seasons, expanded spatial coverage, and concurrent atmospheric monitoring to better constrain the relative contributions of local and large-scale transport processes to MP contamination in remote freshwater systems.

## 5 Conclusion

This study provides the first comparative baseline assessment of microplastic contamination in remote mountain lakes across two contrasting regions of the Americas, contributing to a growing but still limited body of evidence on MP pollution in pristine freshwater systems of the Southern Hemisphere and western North America. MPs were detected in all 15 lakes sampled, with uniformly low but ubiquitous concentrations (mean: 0.75 ± 0.62 particles m^-3^) regardless of geographic region, socioeconomic context, or degree of anthropogenic influence. The dominance of PES and PET fibers, together with the prevalence of dark and vivid-colored particles, points to outdoor recreation and synthetic textile use as key diffuse sources in these environments.

None of the proposed hypotheses relating MP concentrations to local predictors were supported. The absence of significant relationships with watershed characteristics, lake morphometry, altitude, proximity to urban centers, or public access suggests that local factors alone do not govern MP contamination in truly remote settings. Instead, the uniform MP signature observed across both regions, despite their contrasting socioeconomic contexts, points to large-scale diffuse transport mechanisms, particularly atmospheric deposition, as the dominant delivery pathway.

Specific lakes associated with higher tourism pressure, solid waste infrastructure, or hydrological connectivity exhibited elevated concentrations, indicating that localized anthropogenic influences can still modulate MP levels at the site scale.

These findings carry important implications for monitoring and policy. Remote and protected areas cannot be assumed free of MP contamination and should be incorporated into national and international freshwater monitoring frameworks, ideally as sentinel sites for tracking long-term contamination trends. Effective mitigation will require coordinated action at regional and global scales, addressing diffuse sources such as atmospheric transport and synthetic textile use, as discussed in ongoing global plastic treaty negotiations (Thompson et al., 2024). Future research should prioritize repeated sampling across seasons, concurrent atmospheric deposition monitoring, and expanded geographic coverage to better constrain the relative contributions of local and global transport processes. (Barrows et al., 2017; Blue Marble Geographics, 2014; Koelmans et al., 2019; Li et al., 2018; Google Earth Engine, 2023).

## Data Availability

The original contributions presented in the study are included in the article/Supplementary Material, further inquiries can be directed to the corresponding author.
