# Supplementary material for: Microplastic contamination in remote mountain lakes of the americas: a baseline assessment from Patagonia and Northern California
Source: Front Toxicol. 2026 May 14;8:1851245. doi: 10.3389/ftox.2026.1851245 (PMC13215649; doi:10.3389/ftox.2026.1851245)
Supplement: Supplementary file 1 [file Supplementaryfile1.docx]

**Supplementary material**

**Microplastic contamination in remote mountain lakes of the Americas: a baseline assessment from Patagonia and Northern California**

María B. Alfonso^1*^, Facundo Scordo^2,4^, Carina Seitz^3,4,5^, Andrés H. Arias^6,7^, Ana C. Ronda^6,9^, Gian M. Mavo Manstretta^6^, Sudeep Chandra^3^, Gerardo M.E. Perillo^6,8^, María C. Piccolo^6,9^.

^1^Center for Ocean Plastic Studies, Research Institute for Applied Mechanics, Kyushu University, 6-1 Kasuga-Koen, Kasuga 816-8580, Japan

^2^University of Nevada, Reno’s Biology Department, Wildfire Technology Laboratory, and Global Water Center, 1664 N Virginia St, 89557, Reno, NV, USA.

^3^University of Nevada, Reno’s Biology Department, and Global Water Center, 1664 N Virginia St, 89557, Reno, NV, USA.

^4^Instituto de Investigaciones en Biodiversidad y Medioambiente (CONICET - UNCOMA), Pasaje Gutiérrez 1415, 8400, San Carlos de Bariloche, Río Negro, Argentina.

^5^Departamento de Geología y Petróleo, Centro Regional Universitario Bariloche (CONICET-UNCo), Quintral 1250, 8400 San Carlos de Bariloche, Río Negro, Argentina

^6^Instituto Argentino de Oceanografía (IADO), Universidad Nacional del Sur (UNS)-CONICET, Florida 8000, Complejo CCT CONICET Bahía Blanca, Edificio E1, B8000BFW, Bahía Blanca, Argentina.

^7^Departamento de Química, Área III Química Analítica, Universidad Nacional del Sur, Avenida Alem 1253, B8000DIC, Bahía Blanca, Argentina.

^8^Departamento de Geología, Universidad Nacional del Sur (UNS), Avenida Alem 1253, B8000CPB, Bahía Blanca, Argentina.

^9^Departamento de Geografía y Turismo, Universidad Nacional del Sur, Bahía Blanca, Buenos Aires, Argentina.

# ***S1. Lake’s hydrology***

All the US lakes are located in the Klamath Mountains of Northern California. Lake Picayune (US_PIC; 1860 m.a.s.l.) is a throughflow lake with both inflow and outflow, operating independently from the other lakes in the area. The remaining US lakes are part of the upper basin of the South Fork of the Sacramento River. Lake Gumboot (US_GUM; 1850 m.a.s.l.) receives inflow from Lake Upper Gumboot, a small tributary, and has an outflow to the South Fork of the Sacramento River, classifying it as a throughflow lake. Lake Cliff (US_CLI; 1770 m.a.s.l.) is also a throughflow lake, with inflow from Lake Upper Cliff and three small tributaries, and an outflow that connects to the South Fork of the Sacramento River. Finally, Lake Siskiyou (US_SIS; 970 m.a.s.l.), located near Mount Shasta, is a reservoir formed by Box Canyon Dam on the Sacramento River. It has both inflow and outflow through the Sacramento River, with several small tributaries contributing to its hydrology.

The lakes in Argentine Patagonia are interconnected within the Futaleufú River Basin, with each lake having distinct hydrological features and connections to various rivers that finally drain through Chile to the Pacific Ocean. At the upper basin are Lake Cholila (AR_CHO) and Lake Pellegrini (AR_PEL), whose affluents connect to the Carrileufú River. The Carrileufú River is the main tributary of Lake Rivadavia (AR_RIV), whose outflow is the Rivadavia River. The Rivadavia River is the main tributary of Lake Verde (AR_VER), whose outflow is the Arrayanes River. The Arrayanes River ends in Lake Futalaufquen (AR_FUTA), which also receives water from the emissary of the shallow Lake Larga (AR_LAR). AR_RIV, AR_VER, AR_FUTA, and AR_LAR are located within Los Alerces National Park. Lake Futalaufquen flows into Lago Krüger, whose emissary is the Frey River, one of the main tributaries of the Futaleufú Reservoir (AR_DFUT), whose outflow is the Futaleufú River. Another main tributary of the Futaleufú River is the Corintos River, which receives water from tributaries that are outflows of the remaining Argentine lakes located outside the National Park boundaries. The shallow lakes Zeta (AR_ZET) and Willimanco (AR_WIL) both connect to the Esquel River, which after crossing the cities of Esquel and Trevelin merges with the Corintos River from the north. Lake Cronómetro (AR_CRO), feeding into the Río Cronómetro, contributes to the Corintos River system from the west in its mid basin. Lake Rosario (AR_ROS) outflow is the Natifal River, which flows into the Corintos River in its lower basin. These lakes form a complex hydrological network within the Futaleufú River Basin, some of them protected within national park boundaries.

# **Table S1.** Survey details, total microplastics values, and abundance values for particles equal and/or larger than 80 μm (MPs/m^3^) for each lake. Lakes from Argentina are in blue, and those from the United States are in yellow.

| **Lake/ Reservoir** | **Code** | **Date**  **(dd/mm/yyyy)** | **Lat** | **Long** | **Survey method** | **Sample**  **vol. (m^3^)** | **Total MPs** | **MPs/m^3^**  **(≥ 80 μm)** |
| --- | --- | --- | --- | --- | --- | --- | --- | --- |
| Siskiyou | US_SIS | 6/9/2020 | 41.280151 | -122.33266 | trawling | 46.6 | 28 | 0.6 |
| Cliff | US_CLI | 26/7/2020 | 41.199504 | -122.490183 | trawling | 46.6 | 28 | 0.6 |
| Picayune | US_PIC | 27/7/2020 | 41.221979 | -122.523682 | trawling | 25.63 | 71 | 2.8 |
| Gumboot | US_GUM | 27/7/2020 | 41.211246 | -122.51047 | trawling | 46.6 | 34 | 0.7 |
| Rosario | AR_ROS | 19/12/2019 | -42.461697 | -71.325144 | trawling | 32.4 | 36 | 1.0 |
| Futaleufu | AR_DFUT | 20/12/2019 | -42.461697 | -71.658938 | trawling | 32.4 | 12 | 0.3 |
| Verde | AR_VER | 21/12/2019 | -42.461697 | -71.742306 | trawling | 29.16 | 29 | 0.9 |
| Futalaufquen | AR_FUTA | 21/12/2019 | -42.461697 | -71.739452 | trawling | 32.4 | 22 | 0.6 |
| Cronometro | AR_CRO | 22/12/2019 | -42.461697 | -71.072211 | trawling | 32.4 | 15 | 0.4 |
| Zeta | AR_ZET | 23/12/2019 | -42.461697 | -71.350785 | trawling | 32.4 | 9 | 0.2 |
| Willmanco | AR_WIL | 23/12/2019 | -42.461697 | -71.269499 | trawling | 12.96 | 17 | 1.1 |
| Larga | AR_LAR | 24/12/2019 | -42.461697 | -71.562287 | trawling | 32.4 | 11 | 0.3 |
| Rivadavia | AR_RIV | 25/12/2019 | -42.461697 | -71.681597 | trawling | 32.4 | 33 | 0.9 |
| Pellegrini | AR_PEL | 27/12/2019 | -42.461697 | -71.384841 | trawling | 32.4 | 20 | 0.5 |
| Cholila | AR_CHO | 28/12/2019 | -42.461697 | -71.640362 | trawling | 32.4 | 15 | 0.4 |

# **Table S2.** Morphological variables, distance to urban centers (km), public access status, and categories assigned for each lake. Lakes from Argentina are in blue, and those from the United States are in yellow. Maximum depth in US and AR lakes was obtained from U.S. Forest Service (2014), and Quirós & Drago (1985), respectively. *Man-made reservoirs. Acronyms: Lake Rosario (AR_ROS), Futaleufú Reservoir (AR_DFUT), Lake Verde (AR_VER), Lake Futalaufquen (AR_FUTA), Lake Cronómetro (AR_CRO), Lake Zeta (AR_ZET), Lake Willimanco (AR_WIL), Lake Larga (AR_LAR), Lake Rivadavia (AR_RIV), Lake Pellegrini (AR_PEL) and Lake Cholila (AR_CHO),Lake Siskiyou (US_SIS), Lake Cliff (US_CLI), Lake Picayune (US_PIC), and Lake Gumboot (US_GUM).

| **Code** | **Altitude (m)** | **Max Depth (m)** | **Area (km^2^)** | **Watershed area (km^2^)** | **Lake area/ watershed area** | **Distance to urban center (km)** | **Public access** | **Category** |
| --- | --- | --- | --- | --- | --- | --- | --- | --- |
| US_SIS* | 970 | 43 | 1.74 | 350.6 | 0.005 | 4 | Yes | Medium/Intermediate |
| US_CLI | 1770 | 25 | 0.09 | 1.6 | 0.056 | 26 | Yes | Medium/Intermediate |
| US_PIC | 1860 | 8.3 | 0.07 | 2.4 | 0.029 | 31 | No | Small/Shallow |
| US_GUM | 1850 | 4.6 | 0.03 | 5.4 | 0.006 | 25.5 | Yes | Small/Shallow |
| AR_ROS | 650 | 57.4 | 14.5 | 230.6 | 0.062 | 23 | Yes | Medium/Intermediate |
| AR_DFUT* | 480 | 120 | 92 | 4864.7 | 0.019 | 20 | Yes | Large/Deep |
| AR_VER | 515 | 30 | 1.4 | 2052.7 | 0.0007 | 43 | Yes | Medium/Intermediate |
| AR_FUTA | 515 | 168 | 44.6 | 3297.9 | 0.014 | 34 | Yes | Large/Deep |
| AR_CRO | 860 | 14 | 3.04 | 44.2 | 0.069 | 51 | No | Medium/Intermediate |
| AR_ZET | 770 | 11 | 0.65 | 24.5 | 0.026 | 5.2 | Yes | Small/Shallow |
| AR_WIL | 700 | 15 | 0.6 | 19.2 | 0.031 | 11 | No | Small/Shallow |
| AR_LAR | 810 | 34 | 2 | 48.5 | 0.041 | 38 | Yes | Medium/Intermediate |
| AR_RIV | 525 | 147 | 21.7 | 1997.4 | 0.011 | 25 | Yes | Large/Deep |
| AR_PEL | 550 | 14 | 5.1 | 319.0 | 0.016 | 3 | Yes | Medium/Intermediate |
| AR_CHO | 540 | 108 | 17.5 | 602.7 | 0.029 | 17 | Yes | Medium/Intermediate |

# **Table S3.** Summary of statistical comparisons of microplastic concentrations (particles/m³) using Kruskal-Wallis and Mann-Whitney U tests.

| **Variable** | **Factor levels (n)** | **Test used** | **Value** | **p-value** |
| --- | --- | --- | --- | --- |
| Country | AR, US (n = 2) | Mann-Whitney U | U = 12 | 0.215 |
| Lake_area_/Depth | Small/Shallow, Medium/Intermediate, Large/Deep (n = 3) | Kruskal-Wallis | χ² = 1.11 | 0.575 |
| Altitude | Low (<1500 m), High (>1500 m) (n = 2) | Mann-Whitney U | U = 9 | 0.220 |
| Access | Access, No access (n = 2) | Mann-Whitney U | U = 8 | 0.170 |
| Distance | Close (<10 km), Moderate (10–30 km), Far (>30 km) (n = 3) | Kruskal-Wallis | χ² = 1.43 | 0.490 |

# **Table S4.** Spearman correlation matrix for microplastic concentrations and morphological and environmental variables for all studied lakes (n = 15). Bold values indicate significant correlations with p < 0.05.

| Variables | particles/m^3^ | Altitude (m) | Max depth(m) | Area (km^2^) | L_area_/ watershed area | Distance from closest town (km) |
| --- | --- | --- | --- | --- | --- | --- |
| particles/m^3^ | 1 |  |  |  |  |  |
| Altitude (m) | 0.195 | 1 |  |  |  |  |
| Max depth (m) | -0.123 | **-0.716** | 1 |  |  |  |
| Area (km^2^) | -0.364 | **-0.797** | **0.831** | 1 |  |  |
| L_area_/ watershed area | 0.136 | -0.281 | 0.116 | 0.018 | 1 |  |
| Distance from closest town (km) | 0.100 | 0.079 | 0.007 | -0.079 | -0.168 | 1 |

**
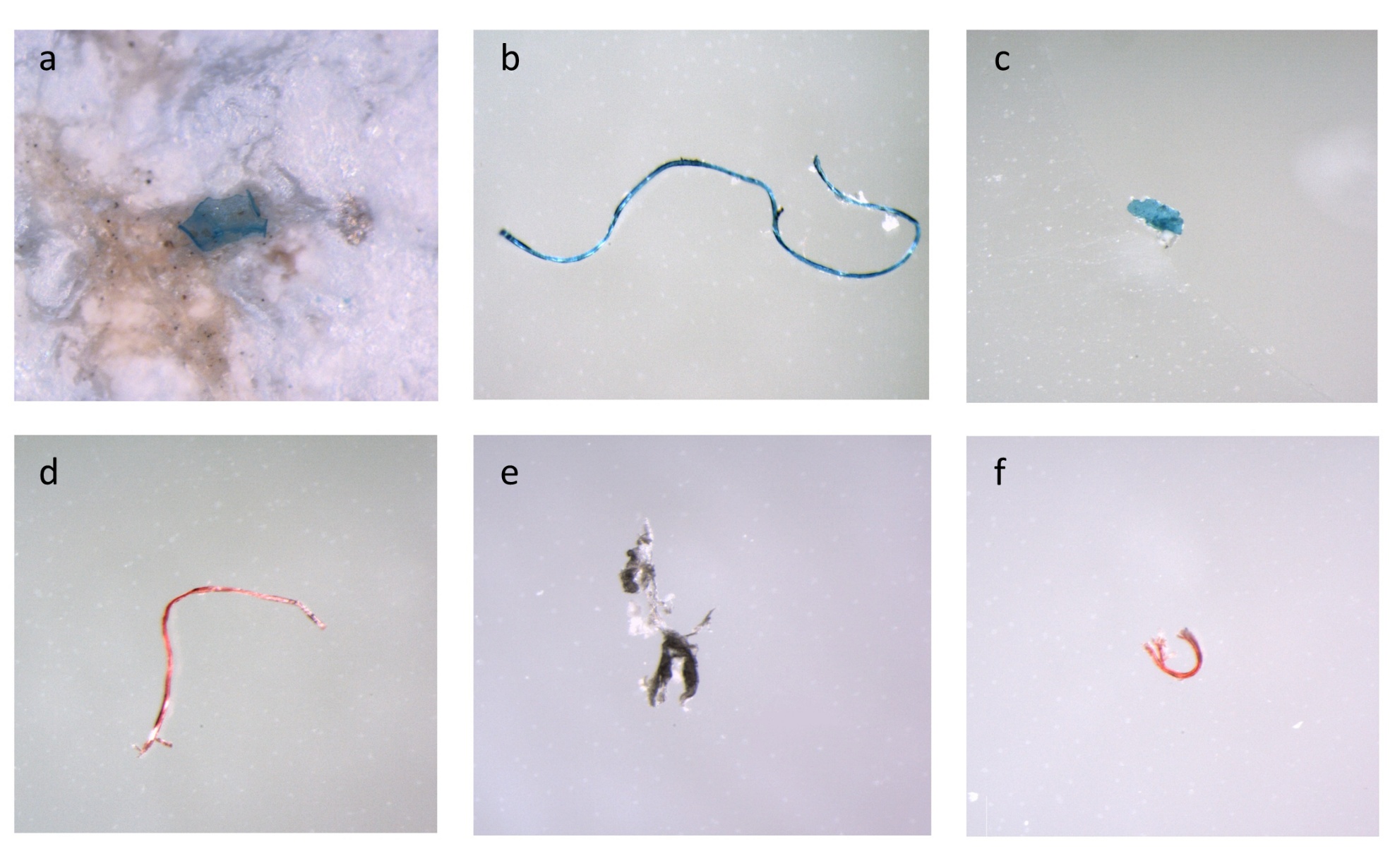
**

# **Figure S1.** Representative examples of microplastics collected from the study sites, including: **(a)** blue film, **(b)** blue fiber, **(c)** blue fragment, **(d)** red fiber, **(e)** black film, and **(f)** red fiber.
